# Enabling supra-aortic vessels inclusion in statistical shape models of the aorta: a novel non-rigid registration method

**DOI:** 10.3389/fphys.2023.1211461

**Published:** 2023-08-10

**Authors:** Martino Andrea Scarpolini, Marilena Mazzoli, Simona Celi

**Affiliations:** ^1^ BioCardioLab, Bioengineering Unit, Fondazione Toscana G. Monasterio, Ospedale del Cuore, Massa, Italy; ^2^ Department of Industrial Engineering, University of Rome “Tor Vergata”, Roma, Italy; ^3^ Department of Information Engineering, University of Pisa, Pisa, Italy

**Keywords:** statistical shape model, non-rigid registration, deformable surface, thoracic aorta, supra-aortic vessels, correspondence optimization

## Abstract

Statistical Shape Models (SSMs) are well-established tools for assessing the variability of 3D geometry and for broadening a limited set of shapes. They are widely used in medical imaging due to their ability to model complex geometries and their high efficiency as generative models. The principal step behind these techniques is a registration phase, which, in the case of complex geometries, can be a critical issue due to the correspondence problem, as it necessitates the development of correspondence mapping between shapes. The thoracic aorta, with its high level of morphological complexity, poses a multi-scale deformation problem due to the presence of several branch vessels with varying diameters. Moreover, branch vessels exhibit significant variability in shape, making the correspondence optimization even more challenging. Consequently, existing studies have focused on developing SSMs based only on the main body of the aorta, excluding the supra-aortic vessels from the analysis. In this work, we present a novel non-rigid registration algorithm based on optimizing a differentiable distance function through a modified gradient descent approach. This strategy enables the inclusion of custom, domain-specific constraints in the objective function, which act as landmarks during the registration phase. The algorithm’s registration performance was tested and compared to an alternative Statistical Shape modeling framework, and subsequently used for the development of a comprehensive SSM of the thoracic aorta, including the supra-aortic vessels. The developed SSM was further evaluated against the alternative framework in terms of generalisation, specificity, and compactness to assess its effectiveness.

## 1 Introduction

Diagnosis and risk stratification of aortic diseases are primarily based on medical imaging techniques which allow the analysis of the anatomy and structure of the heart and vessels. The aortic aneurysm is a disease characterized by an enlargement of the diameter of the aorta. The maximum aortic diameter is the main criterion to understand whether an elective repair is needed to avoid fatal complications, such as rupture or dissection (Erbel et al., 2014). In general, anatomical variations contain valuable information for: (i) diagnosis of aortic pathologies (De Nisco et al., 2020); (ii) planning of patient treatment strategies (Santoro et al., 2021); (iii) design of vascular devices for treatment, e.g., stents-grafts (Lortz et al., 2018); (iv) patient-specific computational analysis (Capellini et al., 2018; Pham et al., 2022); (v) mechanobiological investigation (Mousavi et al., 2021; Vignali et al., 2021). Given their high information content, quantitative analysis of anatomical variations is crucial. Focusing our attention on the thoracic aorta, quantifying its shape and shape variations is difficult due to its morphological complexity and non-in-plane geometry (S-shape morphology) (Donazzan et al., 2014). In current clinical practice, simplified 2d geometrical biomarkers are used to assess pathologies. One example is during aortic aneurysm prognosis, for which the maximum diameter measurement is the most common 2d biomarker used (Erbel et al., 2014). However, it is widely thought that this method has low prognostic significance since it fails to capture the full three-dimensional geometric variability that allows a higher comprehension of the disease (Celi and Berti, 2014; Celi et al., 2023). Moreover, the maximum diameter does not exploit the abundance of information contained in the current tomographic imaging techniques available in clinics, e.g., computed tomography (CT) and magnetic resonance (MR) (Celi et al., 2017). Recent approaches based on radial bases function techniques have been proposed to cope with the enlargement of the aneurysm during its progression or during the cardiac cycle (Capellini et al., 2018; Biancolini et al., 2020; Capellini et al., 2021; Calò et al., 2023). Despite the promising approach, these studies are limited to the ascending portion of the aorta, do not invest the whole complexity of the aorta and of the supra-aortic branches, and are limited to patient-specific cases. In recent years, statistical shape models (SSMs) have come into play, being a powerful data analysis tool for assessing complex anatomical variations starting from patient-specific cases moving up to a quantitative, population-level analysis (Goparaju et al., 2022). SSMs have been widely used in medical imaging applications, where the shape and possible shape variation of an anatomical structure are derived and quantified from a set of shape instances of the structure. The power of these models lies in their data-driven nature and ability to model biological shapes that have an intrinsic intra-subject high variability and complexity. In addition, the complexity of developing SSMs is directly proportional to the morphometric differences between the shapes under investigation. Indeed, the preliminary phase to create a SSM requires the establishment of a dense correspondence between each point of the shape instances, which is a non-trivial task. In the SSM context, this correspondence problem is commonly addressed using either group-wise approaches, which simultaneously register all shapes to a common reference frame, or pair-wise approaches, which establish correspondences between individual shapes and a selected reference shape (Oguz et al., 2016). With particular attention to the thoracic aorta, the solution to this problem is particularly challenging due to the presence of branch vessels. The latter are characterized by smaller diameters and different starting positions along the arch, which increase shape variations and make the deformation problem a multi-scale issue. Several software platforms (frameworks) exist to solve the correspondence problem relying on different modelling approaches and assumptions. The most widely used are ShapeWorks (Cates et al., 2017), Deformetrica (Durrleman et al., 2014), and *GIAS*_2_ (Zhang et al., 2018). Starting from these frameworks, several SSMs of the thoracic aorta have been proposed by Bruse et al. (2017); Sophocleous et al. (2019); Liang et al. (2018); Du et al. (2022); Thamsen et al. (2021) and Bruse et al. (2016). However, all these studies are characterized by significant geometrical simplifications, such as considering the aorta as a curved tubular vessel without including supra-aortic vessels. These branches are of fundamental importance not only for the physio-pathological aspects, but also because their position and orientation angle are crucial pieces of information for the design and evaluation of thoracic endografts with branched components. Moreover, from the computational fluid dynamic (CFD) point of view, they allow the definition of the boundary flow conditions (Boccadifuoco et al., 2016; 2018; Numata et al., 2016), so their removal represents an important limitation for these applications. In order to take into account the role of the supra-aortic vessels for CFD simulations, in Thamsen et al. (2021), the SSM was limited only to the centerline coordinates and the correspondent maximum diameters, and then, the aorta and the supra-aortic vessels were approximated to tubular (axial-symmetric) surfaces.

This paper presents a novel non-rigid registration algorithm, able to accurately solve the correspondence problem considering the whole aorta and the supra-aortic vessels. This algorithm optimizes a differentiable distance function through a modified gradient descent with a combinatorial Laplacian regularization term, which allows the inclusion of specific constraints in the objective function. Specifically, the following innovations were introduced compared to previous work, which allowed a better solution of the correspondence problem for the entire thoracic aorta with the inclusion of supra-aortic vessels:

• Integration of the regularization term within the optimization process, rather than typical addition to the objective function.
• Introduction of a multi-scale approach in the registration phase.
• Introduction of open-boundaries as landamarks to achieve a proper registration of the supra-aortic vessels.

Our method is firstly described and then tested on a dataset of healthy and aneurysmatic thoracic aortas.

## 2 Theoretical background

In this section, we briefly introduce the key concepts related to the proposed SSM algorithm, with particular reference to the metric (*d*) associated to the objective function (*ϕ*), its minimization and the regularization technique to enhance the convergence of the solution. In the context of shape analysis, a manifold is discretized by a polygonal mesh (M) . This mesh is characterized by a set of points *v* ∈ *V* and edges *e* ∈ *E*: M=(V,E) where *V* contains all the vertices of each polygon (nodes of the mesh), and *E* contains the set of edges *e*_*ij*_ connecting the vertices *v*_*i*_ *v*_*j*_. Each vertex *v*_*i*_ ∈ *V* is described by its spatial coordinates $x_{i}\in R^{3}$ . The non-rigid registration process requires two manifolds: a source $\left(M_{s}\right)$ and a target $\left(M_{t}\right)$ polygonal mesh. In this context, the problem of registration can be defined as the minimization of an objective function $\phi \left(M_{s},M_{t}\right)$ which quantifies the distance between $M_{s}$ and $M_{t}$ , as in Eq. 1:

$$\underset{x_{s}\in V_{s}}{min}\phi \left(M_{s},M_{t}\right)$$ (1)

where **x**_*s*_ ∈ *V*_*s*_ are the Cartesian coordinates of $M_{s}$ .

### 2.1 Chamfer distance

Chamfer Distance (CD), $d_{CD}\left(M_{s},M_{t}\right)$ , is a commonly used loss function in learning-based reconstruction networks (Lu et al., 2022). It has been chosen as descriptive metric for *ϕ*, Eq. 2:

$$\begin{array}{ll} d_{CD}\left(M_{s},M_{t}\right) & =\frac{1}{\left|V_{s}\right|}\underset{x_{s}\in V_{s}}{\sum }\underset{x_{t}\in V_{t}}{\min}\|x_{s}-x_{t}{\|}_{2}^{2}\\ & \;+\frac{1}{\left|V_{t}\right|}\underset{x_{t}\in V_{t}}{\sum }\underset{x_{s}\in V_{s}}{\min}\|x_{s}-x_{t}{\|}_{2}^{2} \end{array}$$ (2)

where **x**_*t*_ ∈ *V*_*t*_ are the Cartesian coordinates of $M_{t}$ , and |*V*_*s*_| denotes the cardinality of the set *V*_*s*_, i.e., the number of vertices. This metric works in two steps: firstly, it computes the distance between a point on one mesh and the nearest point on the other mesh and, finally, averages all of them. The choice of CD is due to its two main advantages: it is simple to implement and it is differentiable (once nearest neighbours are established). It is important to note that $d_{CD}\left(M_{s},M_{t}\right)$ is defined between point clouds *V*, not on the whole mesh surface M=(V,E) .

### 2.2 Gradient descent-based registration

The minimization problem is solved by using a gradient descent-based algorithm, which, in its simplest implementation, is:

$$x_{s}\leftarrow x_{s}-\eta \frac{\partial \phi }{\partial x_{s}}$$ (3)

where *ϕ* has to be a differentiable function and *η* is the learning rate which governs the amplitude of steps during optimization. Performing the registration using simple gradient descent to minimize the objective function *ϕ*, as in Eq. 3, introduces some issues yielding a final tangled mesh with multiple inverted elements, such as turning parts of the mesh inside-out and local self-intersections. In order to address this problem, a smoothness regularization term (Ψ) is commonly added in the objective function in order to penalize irregular deformations.

$$x_{s}\leftarrow x_{s}-\eta \frac{\partial \left(\phi +\Psi \right)}{\partial x_{s}}$$ (4)

### 2.3 Combinatorial Laplacian

The Laplacian operator Δ*f* = ∇ ⋅∇*f* is a well-established differential operator which arises in countless physical problems. One of its most important properties is that it can be used to quantify the overall smoothness of a function on a domain Ω. The Dirichlet energy is a measure of how variable a function is and its formulation is reported in Eq. 5. Given a function *f* on a domain Ω, the energy *E*(*f*) measures how much the function *f* changes over Ω. A constant function, for instance, will have zero Dirichlet energy, while a wildly changing function will have higher values of *E*(*f*). Let us define the Dirichlet energy of a function *E*(*f*) and apply the first Green’s identity:

$$E\left(f\right)=\frac{1}{2}\int_{\Omega }\;\|\;\nabla f\;{\|}^{2}\;dx^{3}=C-\frac{1}{2}\int_{\Omega }\;f\;\Delta f\;dx^{3}$$ (5)

where *C* is a constant which depends only on the values of *f* and ∇*f* on the boundaries of the domain Ω. It follows that the smoothness of a function *f* is inversely proportional to the inner product ⟨*f*, Δ*f*⟩. In the context of manifolds, a generalized discrete Laplacian operator *L* can be defined to operate on surface meshes (Bern and Plassmann, 2000) according to the formulation reported in Eq. 6:

$$L_{ij}\left\{\begin{array}{cc} -w_{ij},\;if\;\;e_{ij}\in E & \\ \sum_{k\in N\left(i\right)}^{}w_{ik}=\deg\left(i\right),\; & if\;\;i=j\\ 0,\; & otherwise \end{array}\right.$$ (6)

where deg(*i*) is the number (N(i)) of neighbours of node *i* − th. The weights $w_{ij}\in R$ discretize the first derivative along an edge, while the sum of multiple first derivatives within *L* resembles a discretization of the second derivative of signals on M . Various weights *w*_*ij*_ can be chosen; when the operator is based only on the topology of the input graph, *w*_*ij*_ = 1 for all edges *e*_*ij*_ ∈ *E* and the operator is called combinatorial Laplacian (Botsch and Kobbelt, 2004; Nealen et al., 2006). In the following, the operator *L* will be referred to as the combinatorial Laplacian. Similarly to Eq. 6, using *L*, a discretized version of the Dirichlet energy can be defined for signals *f* on surface meshes *E*(*f*) = ⟨*f*, *Lf*⟩. This term is conventionally used to build the regularization terms Ψ inside the objective function (Eq. 4) (Nicolet et al., 2021). Despite this, a different approach will be adopted in this work. Rather than including extra terms in the objective function, the Laplacian operator will be used to smooth out the irregularities from the gradient of the objective function, as better detailed in the following section (Subsection 3.2).

## 3 Materials and methods

**FIGURE 1:**
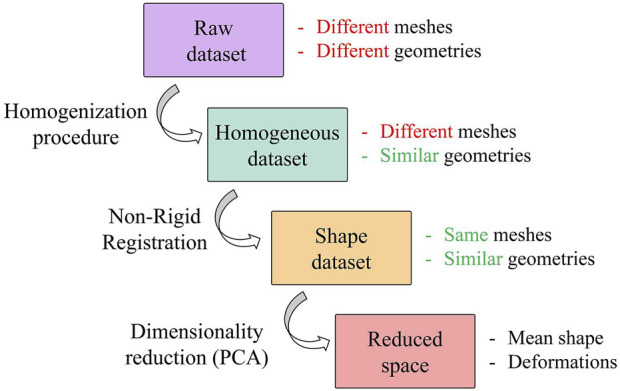
Workflow of the procedure followed to generate the SSM. Focus on datasets with a representation of their key features.

A population of 47 patients (15 females and 32 males; age 65.7 ± 13 years) was considered for this study; 26 were scheduled for surgical thoracic aortic aneurysm treatment, while the remaining 21 were control subjects not affected by aortic diseases. For the aneurysmatic patients, the CT images corresponding to the last radiological follow-up available prior to a recommendation for interventional treatment were considered. The CT scans were performed with a 320-detector scanner (Toshiba Aquilion One, Toshiba, Japan), using iodinated contrast medium (an average pixel size of 0.625 mm, slice thickness of 0.5 mm). Starting from this population, four different datasets are defined and reported in Figure 1.

The *raw dataset* is obtained from a semi-automatic segmentation process performed in 3DSlicer (Fedorov et al., 2012) with an initial refinement in MeshMixer software (Autodesk, 2018) in order to keep the length of the supra-aortic vessel of about 30 mm from the arch surface. It is worth noting that these meshes are affected by several issues, caused by the specific CT scan settings and by the segmentation process. These issues make the meshes unsuitable for the generation of a reliable SSM. A homogenization process is therefore a necessary and fundamental step before the application of the automatic registration algorithm and the subsequent development of the SSM. Specifically, our homogenization procedure involves both discretization and geometrical aspects. Regarding the discretization, meshes with similar initial resolution will be obtained through a remeshing process to a common vertex density (number of vertices of the mesh per surface area); while geometric aspects refer to the fact that each aorta must be aligned to a common reference system (removing translations or rotations) and that aortic branches are clipped at approximately similar lenghts to define a corresponding mapping. This process allows to build a correct parametrization of the shape by factoring out every extrinsic feature of each shape.

### 3.1 Homogenization procedure

**FIGURE 2:**
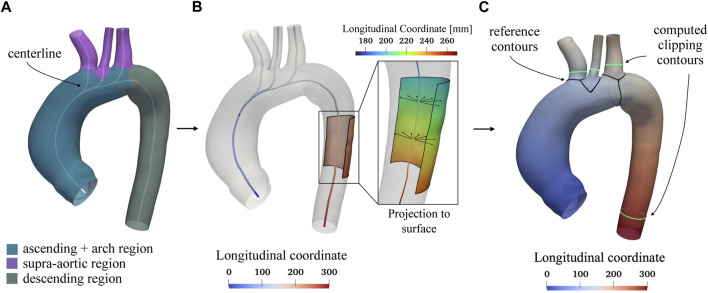
Selected template geometry and longitudinal coordinate system based on the centerline. In Panel **(A)** the different regions of the thoracic aorta, including the ascending, arch, supra-aortic, and descending portions, are displayed with the computed centerline shown in white. The centerline is utilized to establish a longitudinal coordinate system, depicted in Panel **(B)**. This reference system is projected from the centerline onto the aortic surface, as shown in panel **(C)**, and is used to determine specific lengths for clipping the branch vessels. In Panel **(C)**, the black contours at the beginning of each region serve as references for measuring fixed lengths *L*, after which the green contours are subsequently computed to clip each vessel.

As stated above, the homogenization procedure involves geometrical aspects, but it also requires the definition of a template geometry. To minimize the initial distance from each aorta, the template geometry must be enstablished as the “average” geometry in terms of size and shape within the raw dataset. Given that a pair-wise approach will be employed to construct the SSM, the average geometry is crucial for facilitating the non-rigid registration phase required to establish point correspondence between each shape. Due to its importance, the template geometry creation follows an iterative approach within the overall procedure. In the first iteration, a qualitatively selected “average” shape is used to obtain an initial correspondence mapping estimate. The resulting correspondence is then utilized to compute a new average geometry and then to repeat the correspondence optimization. The iterative process concludes when the average geometry converges to a stable shape (Euclidean distance less than 0.1 mm). Figure 2 depicts the first qualitatively selected template, with indications of the four main districts of a thoracic aorta (left part of the figure). For the template geometry, the surface area *A*_*t*_ and the centerline have been computed as references.

In order to homogenize the geometry of each model, the following steps are applied:

1. Surface area (*A*) calculation.
2. Cluster-based remeshing at 35,000(*A*/*A*_*t*_) vertices.
3. Centerline and longitudinal coordinate system computation.
4. Vessels clipping.
5. Cluster-based remeshing at 20,000(*A*/*A*_*t*_) vertices.

Regarding the cluster-based remeshing (Valette et al., 2008) a number of vertices equal to *n* = 35,000 ⋅ *A*/*A*_*t*_ was selected for each aorta in order to guarantee the same average edge length. The centerline (Antiga, 2002), the associated longitudinal coordinate system, and the boundaries marking the separation of the various branches (black lines, Figure 2C) were computed for each branch of the geometry using the VMTK library (Antiga et al., 2008). An example of a centerline is shown in Figure 2 for the template geometry. The longitudinal coordinate system of the centerline was projected on the walls (Figure 2B) and then, exploited in custom scripts, to clip the descending aorta and the three supra-aortic vessels to a fixed length *L* equal to 140 mm and 18 mm, respectively, for each geometry of the *raw dataset*. This choice was made in order to keep as much length as possible for each clipped vessel, according to the raw geometry with shortest vessels. This clipping process was done by generating the green contours (Figure 2C) considering the locus where the longitudinal coordinate on the surface assumed the predefined values $\tilde{L}=C+L$ . *C* is the average value of the longitudinal coordinate where each branch (supra-aortic vessels and descending aorta) starts. Finally, the centerline and surface area *A* were re-computed for each mesh. In order to homogenize the spatial aspects in terms of translation and rotation, a rigid alignment was also applied with respect to the template geometry. A rigid transformation *T* was computed through the Iterative Closest Point (ICP) algorithm (Besl and McKay, 1992; Zhang, 1994) applied to the centerlines. A final cluster-based remeshing to a fixed number of vertices scaled by surface area *n* = 20,000 ⋅ *A*/*A*_*t*_ is applied. The final result is the *homogeneous dataset* (see Figure 1) characterized by meshes with different connectivity but with homogeneous geometries. To quantitatively analyze the changes induced by the cuts, the lengths $l=\left\langle \tilde{L}-C\right\rangle$ were computed, where ⟨⋅⟩ refers to the average along the open boundary. Specifically, *l* ^*raw*^ and *l* ^*homo*^ of each vessel were computed for the *raw* and *homogeneous dataset*, respectively. Lengths were then normalized separately for each vessel with respect to the *raw dataset*, as:

$$\begin{array}{l} l_{\mathrm{norm}}^{\mathrm{raw}}=\left(l^{\mathrm{raw}}-{\bar{l}}^{\mathrm{raw}}\right)/\sigma^{\mathrm{raw}}\\ l_{\mathrm{norm}}^{\mathrm{homo}}=\left(l^{\mathrm{homo}}-{\bar{l}}^{\mathrm{raw}}\right)/\sigma^{\mathrm{raw}} \end{array}$$

where ${\bar{l}}^{\mathrm{raw}}$ and *σ* ^*raw*^ are the mean and standard deviation, respectively.

### 3.2 Non rigid registration algorithm

**FIGURE 3:**
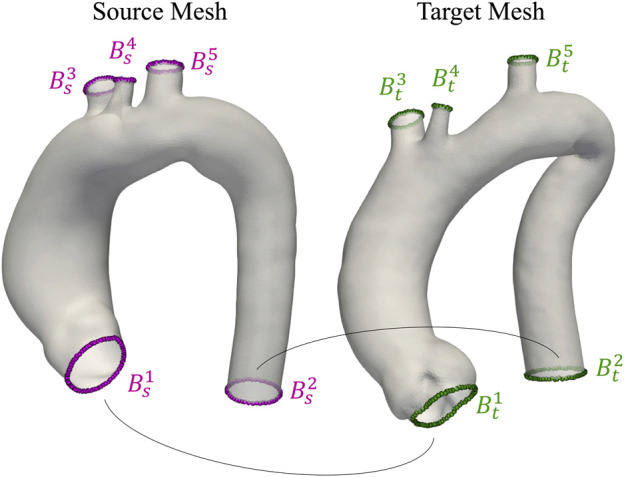
Visualization of two aortas from the homogeneous dataset. Open boundaries *B* ^i^_s_ and *B* ^i^_t_ used as anatomical landmarks during registration are highlighted in purple and green for source and target meshes, respectively.

As defined in Section 2, the non-rigid registration process is based on the minimization of an objective function $\phi \left(M_{s},M_{t}\right)$ . In order to enforce the supra-aortic vessel registration, a specific additional term was added as anatomical constraints, according to Eq. 7:

$$\phi \left(M_{s},M_{t}\right)=d_{CD}\left(M_{s},M_{t}\right)+\overset{5}{\underset{j=1}{\sum }}\alpha_{j}d_{CD}\left(B_{s}^{j},B_{t}^{j}\right)$$ (7)

where $B_{s}^{j}$ and $B_{t}^{j}$ stand for the set of points on the *j*-th open boundary of the source and target mesh, respectively. The resulting objective function $\phi \left(M_{s},M_{t}\right)$ includes both the distance between the two whole surfaces $d\left(M_{s},M_{t}\right)$ and the distance between pairs of open boundaries $\sum_{j=1}^{5}\alpha_{j}d\left(B_{s}^{j},B_{t}^{j}\right)$ . The coefficients *α*_*j*_ are the specific weights defined for each boundary that take into account the discrepancies between the number of nodes on the *B* ^*j*^ edge and on the whole surface. In order to balance this difference, *α*_*i*_ = 0.1 was set *∀i*. Figure 3 depicts the five open boundaries and the relative correspondences ( $B_{s}^{j}$ - $B_{t}^{j}$ ) as anatomical constraints: the first one *B* ^1^ near the aortic root, the second one *B* ^2^ at the end of the descending aorta, and the remaining *B* ^3^ *B* ^4^ and *B* ^5^ on the open ends of the three supra-aortic vessels. As discussed in Section 2.2, the solution of the minimization problem with a pure gradient descent approach (Eq. 3) results in poor-quality meshes. A preconditioned gradient descent algorithm, described in Nicolet et al. (2021), was implemented to overcome this problem. The new update rule is:

$$x_{s}\leftarrow x_{s}-\eta {\left(1+\gamma L\right)}^{-1}\left(\frac{\partial \phi }{\partial x_{s}}\right)$$ (8)

where *L* is the combinatorial Laplacian operator of the source mesh $M_{s}$ . The gradient calculation of Eq. 8 was implemented using automatic differentiation with PyTorch and PyTorch3D libraries (Paszke et al., 2019; Ravi et al., 2020). The preconditioning matrix ${\left(1+\gamma L\right)}^{-1}$ diffuses the gradient *∂ϕ*/*∂* **x**_*s*_ along the mesh, resulting in smoother and more consistent deformations during the optimization. The coefficient *γ* acts as a regularization parameter: higher values of *γ* prioritize the smoothness of the final mesh (more regular deformations), while smaller values improve the reproduction of fine details of the target mesh. For *γ* = 0 the optimization method corresponds to the standard gradient descent. It is worth mentioning that, since the Laplacian contains second-order derivatives, it can be shown how this new update rule resembles a Newton-like second-order optimization scheme (Nicolet et al., 2021). The choice and control of the learning rate *η* during optimization is made by adding momentum terms using the UNIFORMADAM optimizer (Nicolet et al., 2021). The resulting registration algorithm $\left(R\left(M_{s},M_{t}\right)\right)$ is applied between a source and a target mesh, producing a registered mesh $\left(M_{r}\right)$ , isotopological to $M_{s}$ , but with different coordinates **x**_*r*_:

$$M_{r}=R\left(M_{s},M_{t}\right).$$ (9)

Specifically, $M_{r}$ is the surface mesh with vertex coordinates obtained from the minimization problem defined in Eq. 1.

#### 3.2.1 Multi-scale registration

To increase the registration performances, $R\left(M_{s},M_{t}\right)$ is iteratively applied in a multi-scale fashion: the registration results are subsequently interpolated to a gradually finer surface discretization for a total of *n* different spatial scales. Given two initial polygonal meshes $M_{s}=M_{s}\left(0\right)$ and $M_{t}=M_{t}\left(0\right)$ , *n* − 1 coarser meshes are created by remeshing at a smaller number of points:

$$\begin{array}{l} M_{s}\left(0\right)\to M_{s}\left(1\right)\to \cdot \cdot \cdot \to M_{s}\left(n-1\right)\\ M_{t}\left(0\right)\to M_{t}\left(1\right)\to \cdot \cdot \cdot \to M_{t}\left(n-1\right). \end{array}$$

**FIGURE 4:**
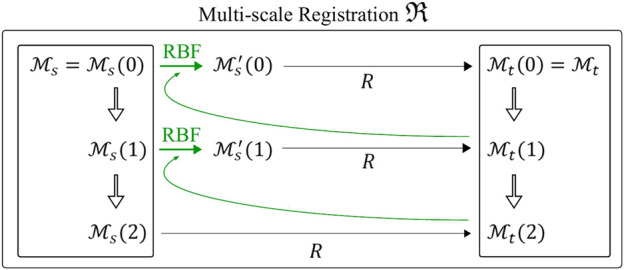
Multi-scale non rigid registration (R) with *n* = 3. First, coarser discretizations of the initial meshes $M_{s}$ and $M_{t}$ are created (inner black boxes). *R* is first applied to the coarsest meshes $M_{s}\left(2\right)$ and $M_{t}\left(2\right)$ on the bottom. Then, the resulting displacement field is interpolated through RBF and applied to the finer source mesh $M_{s}\left(1\right)$ to obtain $M_{s}^{\prime }\left(1\right)$ . The process is repeated until the finest scale, i.e., the original resolutions $M_{s}$ and $M_{t}$ .

As depicted in Figure 4, the registration is initialized between the coarsest meshes $M_{s}\left(n-1\right)$ and $M_{t}\left(n-1\right)$ . The first registered mesh $M_{r}\left(n-1\right)=R\left(M_{s}\left(n-1\right),M_{t}\left(n-1\right)\right)$ is obtained and the deformation field is computed by interpolating the displacement of each point from $M_{s}\left(n-1\right)$ to $M_{r}\left(n-1\right)$ using radial basis functions (RBF). The resulting deformation field is applied to $M_{s}\left(n-2\right)$ to obtain $M_{s}^{\prime }\left(n-2\right)$ . At this point, the registration at the next refinement level brings to $M_{r}\left(n-2\right)=R\left(M_{s}^{\prime }\left(n-2\right),M_{t}\left(n-2\right)\right)$ . This process is repeated until the last registration at original resolution $M_{r}\left(0\right)=R\left(M_{s}^{\prime }\left(0\right),M_{t}\left(0\right)\right)$ is obtained.

The final result of the multi-scale non rigid registration R between the source mesh $M_{s}$ and the target one $M_{t}$ will be:

$$M_{r}=M_{r}\left(0\right)=R\left(M_{s},M_{t}\right).$$ (10)

Three spatial scales (*n* = 3) were considered, resulting in two remeshing steps with 4,000 and 18,000 points for the coarser meshes $M_{s}\left(2\right)$ / $M_{t}\left(2\right)$ and $M_{s}\left(1\right)$ / $M_{t}\left(1\right)$ , respectively. This configuration was selected following a fine-tuning phase that indicated no significant improvements for *n* > 3. Different values of *γ* and *η* were chosen for each resolution level, as detailed in Table 1. The gradual decrease of the regularization parameter *γ* is justified by the multi-scale nature of the registration. At larger scales (when the number of surface points is reduced), the deformation field must capture the target shape’s large-scale features, while at smaller scales, finer details need to be registered, necessitating a lower *γ* value.

**TABLE 1:** Parameters for the *n* = 3 different spatial scales of the non-rigid registration algorithm *R*.

# Points	N	*γ*	*η*
4,000	700	120	0.007
18,000	1,000	80	0.01
20,000	1,500	50	0.02

“# Points” indicates the number of points of the source and target meshes at the corresponding spatial resolution. *N* is the number of iterations, *γ* the regularization factor and *η* the initial learning rate.

#### 3.2.2 Shape dataset creation

Shape is defined as a property which does not change under similarity transformations, i.e., it is invariant to translation, rotation and scaling. In general, shape changes induced by these global transformations should not be modelled by a SSM to keep the model as specific as possible. Concerning our initial dataset, the size variation of the aorta was not considered a similarity transformation (scaling) because it is an integral part of the anatomical variability. In order to create a SSM, all the 3d models has to be represented by the same number of *k*-points and the same points correspondence. The shape **s** is the vector where the coordinates of the *k*-points are concatenated:

$$s={\left(x_{1},y_{1},z_{1},\ldots ,x_{k},y_{k},z_{k}\right)}^{T}$$ (11)

The multi-scale non-rigid registration algorithm described in 3.2.1 was applied between the source $M_{s}$ template geometry and each model of the homogeneous dataset $M_{t}^{i}$ (*i* = 1, …, *M* with *M* = 47 the total number of aortas). This allowed (i) to correctly distribute the elements of **s** on each surface model, (ii) to create a dataset of shapes with the same connectivity *E* and (iii) to obtain a set of isotopological surface meshes $M_{t}^{i}$ where:

$$M_{r}^{i}=R\left(M_{s},M_{t}^{i}\right)$$ (12)

Finally, a generalized procrustes alignment (GPA) (Goodall, 1991; Heimann and Meinzer, 2009) was performed to describe the pure shape of each aorta by the removal of any possible bias introduced by the selection of the source template $M_{s}$ . The GPA aligns a set of shapes **s**_*i*_ to their unknown average $\bar{s}$ by iteratively applying a rigid transformation to each shape of the dataset to minimize the distance from $\bar{s}$ , which changes at each iteration.

$$\bar{s}=\frac{1}{M}\overset{M}{\underset{i=1}{\sum }}s_{i}$$ (13)

The procedure ends when the maximum difference between the coordinates of $\bar{s}$ from one iteration to the next is less than 0.001 mm. The resulting set of shapes constitutes the *shape dataset*. In order to assess the similarity between the obtained *shape dataset* and the related target shapes, both the Chamfer (Eq. 2 and Hausdorff distance (Eq. 14) were computed. The Hausdorff distance is defined as:

$$\begin{array}{ll} d_{H}\left(M_{s},M_{t}\right) & =\text{max}\left(\underset{x_{s}\in V_{s}}{\max}\underset{x_{t}\in V_{t}}{\min}\|x_{s}-x_{t}{\|}_{2},\right.\\ & \left.\;\;\underset{x_{t}\in V_{t}}{\max}\underset{x_{s}\in V_{s}}{\min}\|x_{s}-x_{t}{\|}_{2}\right) \end{array}$$ (14)

In order to make the two distances comparable, the square root of the Chamfer distance was considered because of the quadratic terms in its definition (Eq. 2). These distances are two of the most popular evaluation criteria to compare the similarity between different point clouds (Urbach et al., 2020; Lu et al., 2022). Both metrics are based on the nearest neighbours; while the Chamfer uses the average values, the Hausdorff metric works with the maximum ones. Moreover, to provide insights into the robustness of the algorithm, a stability analysis was carried out by introducing small random perturbations to the surface node coordinates of the same target geometry. This analysis is reported in the “Sensitivity study on target geometry” section of the Supplementary Material.

### 3.3 Dimensionality reduction

Dimensionality reduction is the final step in constructing a SSM. Principal Component Analysis (PCA) is the most widely used algorithm in the context of SSMs, as it (i) reduces the dimensionality of a dataset, (ii) enhances its interpretability, and (iii) minimizes information loss. PCA calculates the eigendecomposition of the covariance matrix *S* of the *shape dataset* as follows:

$$S=\frac{1}{M-1}\overset{M}{\underset{i=1}{\sum }}\left(s_{i}-\bar{s}\right)\left(s_{i}-\bar{s}\right)$$ (15)

Subsequently, PCA extracts the *m* = min((*M* − 1), 3*k*) principal modes of variation (eigenvectors *ϕ*_*i*_) and their associated variances (eigenvalues *λ*_*i*_). The dimensionality reduction of the SSM can be further optimized by limiting the number of modes to the first *m*′ < *m* eigenvectors, as these are ordered by decreasing values of variance (*λ*_1_ ≥…≥ *λ*_*m*_). The optimal *m*′ value is typically made by analyzing the percentage of cumulated variance:

$$\Lambda_{i}=\frac{\sum_{j=0}^{i}\lambda_{j}}{\sum_{j=0}^{m}\lambda_{j}}$$ (16)

When a satisfactory cumulated variance value is achieved, e.g., when Λ_*m*′_ = 99%, the number of modes can be truncated and the initial dataset can be represented in a considerably more compact manner without significant information loss. Finally, new shapes can be created by varying the coefficients *ω*_*i*_, for which a Gaussian distribution is assumed, of the linear combinations:

$$s=\bar{s}+\underset{i}{\sum }\omega_{i}\phi_{i}$$ (17)

To intrinsically evaluate a SSM, three key metrics are commonly employed (Davies, 2002; Ericsson and Karlsson, 2007): generalisation *G*(*K*), specificity *S*(*K*), and compactness *C*(*K*). The variation of these metrics with respect to the number of employed modes (*K*) provides valuable insights into the quality of the dimensionality reduction. Generalisation measures the SSM’s ability to accurately reconstruct shapes not included in the training dataset through leave-one-out cross validation. Specificity assesses the SSM’s capability to generate shapes that are solely representative of the original dataset’s shape variability; while compactness evaluates the efficiency of the SSM by determining the minimal number of principal components required to represent a given percentage of the total shape variability. The generalisation metric is defined as the mean squared leave-one-out reconstruction error

$$G\left(K\right)=\frac{1}{M}\overset{M}{\underset{i=1}{\sum }}\epsilon_{i}^{2}\left(K\right)$$ (18)

where $\epsilon_{i}^{2}\left(K\right)$ is the reconstruction error for shape *i* (the one left outside the training dataset) using only the first *K* modes. The leave-one-out cross validation was computed by creating *M* reduced datasets, with *M* − 1 meshes each (meshes from the *shape dataset*), excluding a different sample from time to time. *M* reduced SSMs were therefore generated out of these reduced datasets and each of these reduced SSMs was used to reconstruct the corresponding excluded shape. In order to achieve this, each excluded shape was projected into the relative PCA latent space to compute the eigenvector coefficients *ω*_*i*_. The similarity between the original excluded shape and the resulting approximated one was assessed in terms of the Euclidean distance between the corresponding points. The compactness is defined as the percentage of cumulative variance explained by the model up to a certain number of modes (i.e., the previously defined Λ_*i*_ in Eq. 16)

$$C\left(K\right)=\frac{\sum_{i=1}^{K}\lambda_{i}}{\sum_{j}^{m}\lambda_{j}}$$ (19)

Specificity can be quantified by randomly generating *j* = 1, …, *H* (a large number of) samples from the shape space, using the first K eigenvectors and eigenvalues, assuming a multivariate normal distribution, and computing the Euclidean distance to the closest training sample

$$S\left(K\right)=\frac{1}{H}\overset{H}{\underset{j=1}{\sum }}\epsilon_{j}^{\prime }{\left(K\right)}^{2}$$ (20)

where $\epsilon_{j}^{\prime }\left(K\right)$ is the mean Euclidean distance between the generated shape and its nearest sample of the training set (point correspondence is already defined). Specifically, $\epsilon_{j}^{\prime }\left(K\right)=\underset{i}{min}\left(\frac{1}{P}\sum_{h=1}^{P}\|x_{j}^{h}\left(K\right)-x_{i}^{h}\left(K\right){\|}_{2}\right)$ , where *P* is the number of vertices in the two meshes, $x_{i}^{h}\left(K\right)$ are the coordinates of the *h*-th vertex of the *i*-th shape of the *shape dataset* and $x_{j}^{h}\left(K\right)$ are the coordinates of the *h*-th vertex of the *j*-th randomly generated shape. The nearest sample was determined by calculating the mean Euclidean distance between the generated shape and all the samples in the training dataset, and then taking the one that gave the smallest value. If for two SSMs *A* and *B*, *S*_*A*_(*K*) ≤ *S*_*B*_(*K*) for most of *K* values we can conclude that model *A* is more specific than model *B*. The same is true for generalisation, while for compactness a higher value of *C*(*K*) means that the dimensionality reduction was more effective. In the construction of a SSM, a trade-off between these three metrics should be achieved.

In order to evaluate the effect that the selection of different open-boundary constraint (*α* values) has on generalisation, specificity and compactness, the results of a sensitivity analysis with *α*_*j*_ equal to 0.01, 0.1 and 1.0, are included in a dedicated section of the Supplementary Material (Sensitivity study on boundary loss coefficient). Moreover, to evaluate the relative contribution of the two innovative aspects introduced in our algorithm (multi-scale approach and open-boundary constraint), we repeated the whole procedure for generating a SSM with only one of the two contributions active. For each, we then calculated the generalisation, specificity, and compactness metrics and made an extrinsic qualitative assessment. It is worth to notice that the deactivation of the open-boundary constraint corresponds to setting *α*_*j*_ coefficients equal to zero. Whereas, the deactivation of the multi-scale approach, was performed with only one registration *R* by setting the number of points equal to 20,000 (finest resolution), *γ* = 50 and *η* = 0.02. In order to address the slower convergence speed of this version, the number of iterations *N* has been increased from 1,500 to 4,000.

### 3.4 Implementation

The overall statistical analysis and the registration algorithm were implemented in Python 3.8 using PyTorch 1.11 library (Paszke et al., 2019). The latter was used to develop the computationally intensive routines and allowed us to obtain both a CPU (multi-threaded) and GPU version of the algorithm. A Linux workstation running Ubuntu 20.04LTS with an Nvidia GeForce RTX 3090 GPU (24 Gb) was used to develop and execute the code. The most computationally demanding operation of the registration algorithm is the evaluation of the Chamfer distance and its gradient at each iteration. In order to speed up this operation, the PyTorch3d Library was used (Ravi et al., 2020). In our fixed-iteration setting, in which the number of iterations remains constant regardless of the source and target mesh geometries, the registration process between these two meshes, each consisting of 20,000 points, takes approximately 135 s using the hardware specified above (GPU version of the code).

## 4 Results

In this section, the main results of our workflow are reported with particular attention to the obtained homogeneous shape and reduced dataset.

### 4.1 Homogeneous dataset

**FIGURE 5:**
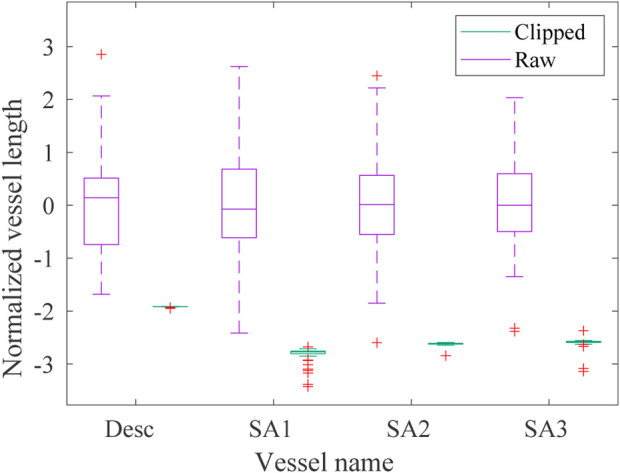
Boxplot of the normalized lengths *l*_norm_ of each vessel (descending aorta *desc* and first, second and third supra-aortic vessel *SA*, respectively) of the *raw* (violet) and *homogeneous* (green) datasets. The green boxes are not clearly visible due to the reduction of the homogeneous lengths range achieved with the clipping procedure.

The effects of the automatic clipping procedure developed to homogenize the lengths of each branch vessel of the dataset are reported in the boxplots in Figure 5 where the statistical distribution of the normalized lengths $l_{\mathrm{norm}}^{\mathrm{raw}}$ and $l_{\mathrm{norm}}^{\mathrm{homo}}$ (before and after the clipping procedure) are depicted. It is worth noticing that the green boxplots associated with the *homogeneous dataset* are not clearly visible due to the clipping procedure that reduces the range in which the length distributions of the homogenized vessels fall in.

### 4.2 Non-rigid registration algorithm

**FIGURE 6:**
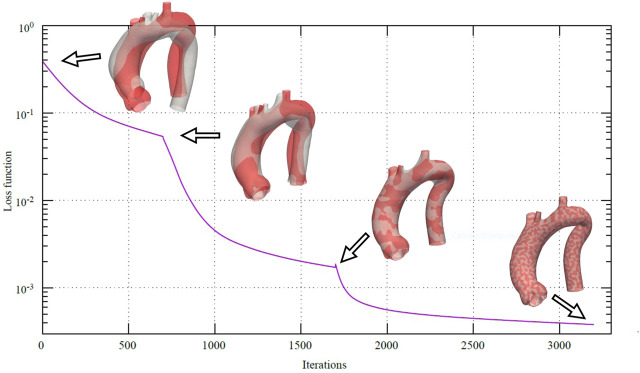
Loss function during the multi-scale non-rigid registration (R) between the template geometry (grey) and a random sample from the homogeneous dataset (red). Changes in slope are due to the related changes in resolution.

In Figure 6, the loss function $\phi \left(M_{s},M_{t}\right)$ during the registration between the template geometry and a random sample from the homogeneous dataset is depicted. An animated visualization of the registration process has been included in the Supplementary Video S1. It is worth noting the abrupt changes in the slope of the loss function as the mesh resolution changes according to the setting reported in Table 1. In fact, when large-scale details are detected, *ϕ* starts to converge; the change in resolution provides an opportunity to capture finer details of the target geometry, and then *ϕ* begins to decrease again.

#### 4.2.1 Shape dataset

**FIGURE 7:**
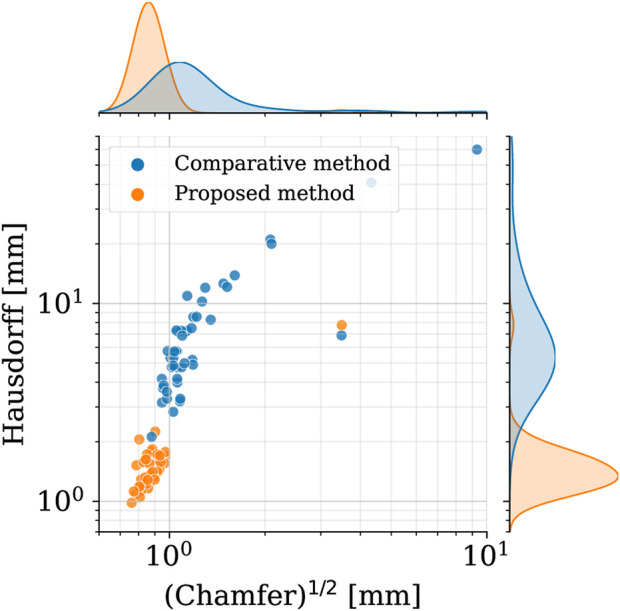
Square root of the Chamfer (*x*-axis) and Hausdorff (*y*-axis) distances between each registered shape (*shape dataset*, i. e., after registration) and its original 3d model (*homogeneous dataset*, i.e., before registration). Results of both the proposed method (orange) and the reference one (blue). In the top and right subplots, the marginal probability density functions of the distances are shown (estimated through kernel density estimation).

**FIGURE 8:**
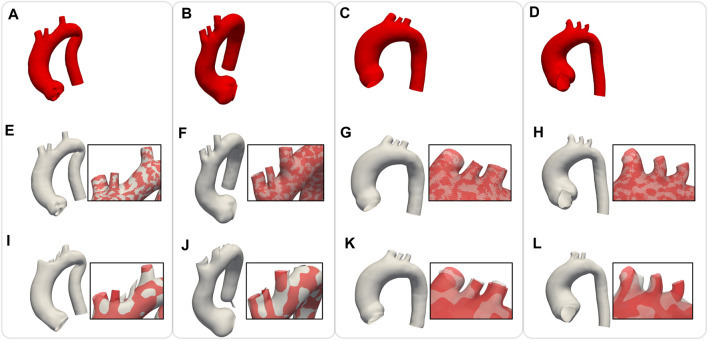
Comparison of non rigid registration between the proposed approach (R) and the reference method. Four representative target geometries of the *homogeneous dataset* **(A–D)**; results of the multi-scale non-rigid registration algorithm, i.e., the proposed approach **(E–H)**; results obtained with the reference method **(I–L)**.

In order to assess the geometric differences between each geometry before and after the registration process, we calculated the Chamfer and Hausdorff distances for each sample within the *homogeneous* and *shape datasets*. Ideally, with infinite mesh resolution, these distances would be zero if the registration-induced deformations did not introduce any geometric artifacts. However, with finite mesh resolution, the minimum value for these distances is of the order of the average edge length of each surface mesh. To further evaluate the performance of our non-rigid registration method, we compared it with the Deterministic Atlas in Deformetrica, a leading statistical shape modeling framework (Goparaju et al., 2022), by processing our *homogeneous dataset* using both methods. This comparison was motivated by the fact that all studies on statistical shape modeling of the aorta that relied on surface correspondence (and not just centerline) utilized this framework (Bruse et al., 2016; Sophocleous et al., 2019; Du et al., 2022). In the following sections, the deterministic atlas method of Deformetrica, which serves as the benchmark for our non-rigid registration algorithm, will be referred to as “the comparative method” or “the reference method” for the sake of simplicity and clarity. Hyperparameter tuning for the comparative method was carried out in order to obtain best results in terms of the metrics that will be later presented in this article. It is important to emphasize that the comparative method was employed according to the methodologies followed by the cited articles. Figure 7 illustrates the values of both distances, along with their estimated probability density functions, for both methods. For our approach, the Chamfer distance consistently shows values below 1 millimeter, which is in accordance with the average edge length of the *homogeneous dataset* (1.2 mm). The averaged distances across all shapes are reported in Table 2. The proposed method shows both smaller and narrower distributions of these two distance metrics. In addition, a qualitative comparison of four random samples is reported in Figure 8. For each sample, the figure shows the original 3d model in the *homogeneous dataset* (Figures 8A–D) and the final registered shapes (with the enstablished point correspondence) for both our method (Figures 8E–H) and the comparative framework (Figures 8I–L). In the magnification boxes, the original geometries (red mesh in transparency) are superimposed to better appreciate the details of the supra-aortic vessels. From subfigures (I) and (J) we can see how the surpa-aortic vessels are completely mismatched (and also the descending aorta presents geometrical artifacts in (J)). On the other hand, for subfigures (k) and (L), supra-aortic vessels are correctly matched, but very small scales features, such as their orientation, are not recovered. The outlined defects reflect the limited flexibility imposed by the fixed kernel size of the deformation field of the comparative method. Precisely because of this, supra-aortic vessels are often incorrectly registered, whereas in the proposed algorithm this problem is solved by the more flexible nature of the deformation field and the addition of open-boundaries constraints in the landmark term of Eq. 7.

**TABLE 2:** Mean ± standard deviation of Chamfer and Hausdorff distance across the whole dataset.

Method	(Chamfer distance)^1/2^ [mm]	Hausdorff distance [mm]
reference method	1.44 ± 1.32	8.73 ± 9.93
proposed method	0.92 ± 0.39	1.56 ± 0.96

### 4.3 Dimensionality reduction

**FIGURE 9:**
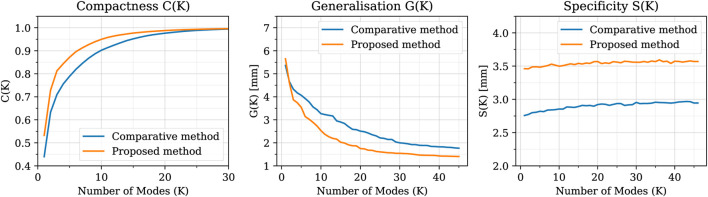
Compactness, Generalisation and Specificity metrics in comparison between the SSMs built using the proposed method (orange) and the comparative one (blue). The proposed method shows better compactness and generalisation metrics, while the comparative method presents a slightly better specificity.

**FIGURE 10:**
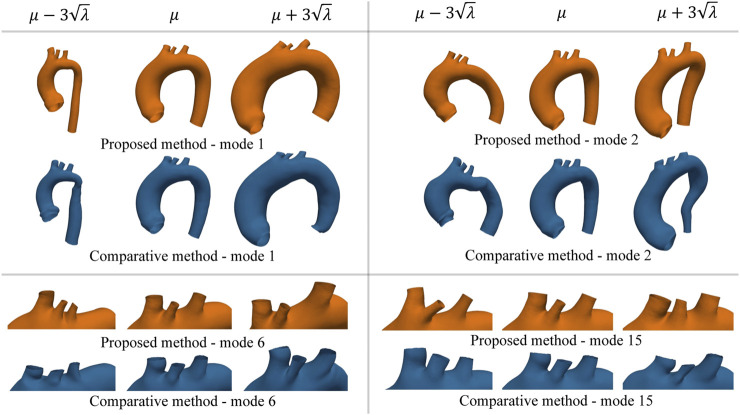
Example of new shapes generated by the SSM of either the proposed or the comparative method through Eq. 17. In each sub-panel, the variation of only one mode was considered by setting all the other modes’ coefficients equal to zero. Extreme shape variations (left ad right side of the mean shape *μ*) were generated by setting the varying coefficient to three times its standard deviation $\omega_{i}=\mu_{i}\pm 3\sqrt{\lambda_{i}}$ . The average shape of the two methods is very similar; while the extreme shapes of the comparative method exhibit geometrical artifacts due to the wrong correspondence mapping of particular regions.

The values of the intrinsic metrics (compactness, generalisation, and specificity) are reported in Figure 9 for both the proposed algorithm and the comparative method. From the compactness analysis, it can be observed that, with our approach, it is possible to reduce the dimensionality of the problem by using only the first 7 modes of the SSM as they explain the 90% of the variability in the dataset (22 modes for 99%). In contrast, using the comparative method, it necessitates 10 modes to explain 90% of the variability (26 modes for 99%). Concerning the generalisation metric, for low values of *K*, the reconstruction error of the two methods is comparable; while for *K* > 4, the proposed approach exhibits superior generalisation properties. Regarding the specificity metric, the results indicate that the comparative method consistently yields a specificity value approximately 0.5 mm lower than our proposed approach. As an extrinsic qualitative assessment of the SSM’s quality it is possible to generate new shapes, by varying the coefficients $\omega_{i}=\pm 3\sqrt{\lambda_{i}}$ (Eq. 17) for *i* = 1, 2, 6 and 15, and assess the anatomical variability captured by the SSM. This analysis is reported in Figure 10 The first modes (*i* = 1, 2) explain most of the variability within the dataset, as they account for most of the variance. On the other hand, considering higher modes (*i* = 6, 15), large-scale details do not vary substantially, while changes are observed in fine details such as the position and the orientation of supra-aortic vessels.

**FIGURE 11:**
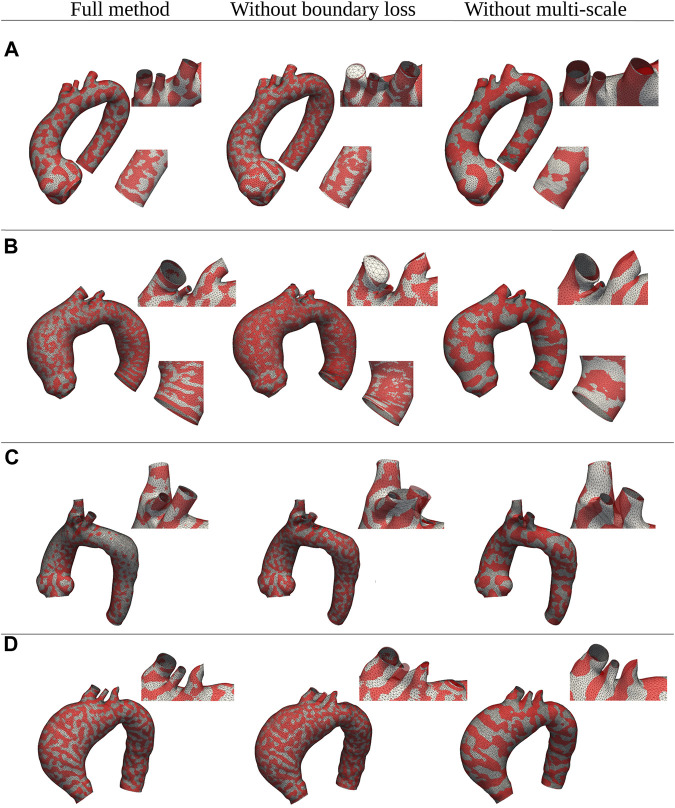
Qualitative comparison between shapes registered with three distinct versions of the proposed method: the full method, the method without the multi-scale feature, and the method without the boundary loss constraint. Four examples of different shapes from the original dataset **(A–D)** are shown in red; while the registered meshes are superimposed in white.

**FIGURE 12:**
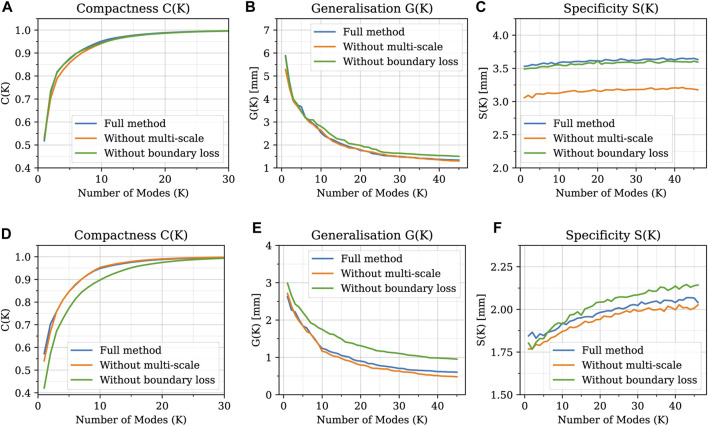
Generalisation, specificity, and compactness metrics measured on SSMs built with the three different methods, both for the complete geometries **(A–C)** and the supra-aortic branches alone **(D–F)**.

Regarding the evaluation of the relative contribution of the two innovative aspects introduced in our algorithm (multi-scale approach and open-boundary constraint), the results in terms of qualitative evaluation and intrinsic metrics are shown in Figures 11, 12, respectively; Figure 11 presents a qualitative comparison of results for four sample aortas from our dataset. An animated visualization of the registrations with the three different methods has also been included in a video in the Supplementary Video S2. The original model is marked in red, overlaid with: 1) our proposed registration, 2) our proposed registration without the open-boundary loss, and 3) our proposed registration without the multi-scale approach. It is evident from this figure that, both the multi-scale method and the open-boundary loss, are necessary to achieve a high-quality final result, depending on the specific case. Specifically, the multi-scale method ensures faster convergence, and a more homogeneous deformation of elements distributed across the entire surface of each aorta. Moreover, the different regularization terms used at each scale allow to match the large-scale geometric features first, and then focus on smaller details. The open-boundary constraint, on the other hand, ensures a precise match between the various supra-aortic branches, which does not occur in its absence when the branches are small and in close proximity. Figure 12, on the other hand, includes the three metrics (generalisation, specificity, and compactness) measured on the three different methods, both for the complete geometries (A–C) and the supra-aortic branches alone (D–F).

## 5 Discussion

In this work, we have presented a novel non-rigid registration algorithm for the development of SSMs able to solve the correspondence optimization problem for complex anatomical structures. The novelty of the algorithm lies in its unique approach of integrating the regularization term within the optimization process, rather than appending it to the objective function. Additionally, by employing the algorithm in a multi-scale manner, the significance of multi-resolution registration and the presence of landmarks is emphasized when dealing with datasets with complex geometries. Here we applied our algorithm to the whole thoracic aorta. To the best of our knowledge, this is the first SSM that exhibits the inclusion of the supra-aortic vessels succeeding in handling the full complexity and 3D variability of the entire thoracic aorta. Specifically, we implemented the method in the context of a real population of both healthy and aneurysmatic subjects, considering different types of dilations within the sinus-tubular junction, the aortic arch and the ascending and descending aorta. Our tool has demonstrated to be able of handling different types of geometric deformation due to its capability to manage complex scenarios. The developed framework holds potential for several applications from both a clinical and computational perspective. From the clinical point of view, crucial for the successful treatment of thoracic aortic diseases is a deep knowledge of the whole anatomy of the aortic arch in relation to the origins of the supra-aortic arteries. This area is affected by several pathologies, such as aneurysms and dissections, which can currently be treated with open surgery, endovascular or hybrid procedures (Pérez et al., 2017; Sengupta et al., 2022). In all cases, the fundamental aspect is to ensure the vascularization of the supra-aortic vessels in order to avoid adverse events such as ischemic strokes, which lead to brain cell damage or death. The size, position and orientation of the supra-aortic vessels are a crucial factor also in hemodynamic numerical simulations, where their cross-sectional area plays a key role in setting the outflow boundary conditions, while their position influences the entire hemodynamic pattern of the thoracic district (Boccadifuoco et al., 2016; 2018; Antonuccio et al., 2021). The developed non-rigid registration method can be applied to any shape, especially to those without evident landmarks, as in the case of the aorta. In this latter case, it has been shown that, when open boundaries are available, they can be efficiently used as artificial landmarks after an accurate pre-processing of the shapes. This procedure could be applied to any vessel-like geometry of arbitrary topology and also to many other anatomical structures where open boundaries are created by clipping the region of interest from the other structures. In addition, the methodology could also be used to study shape modification over time using prospective studies of the same patient, as in Capellini et al. (2018) and Sophocleous et al. (2022). Again, as in the previously mentioned studies, the latter one does not include supra-aortic vessels in the analysis. Besides the analysis of shape variations, a SSM can also be used as a generative model. In order to do this, once the reduced space is created, new realistic shapes can be generated by sampling the probability distribution of each shape coefficient *ω*_*i*_ in the PCA latent space. This is particularly useful in medical applications where the lack of available data often prevents further investigations. In the landscape of the most widely used tools for SMMs, Deformetrica has emerged as the predominant choice due to its power and versatility (Bruse et al., 2016; Bruse et al., 2017; Sophocleous et al., 2019; Goparaju et al., 2022; Sophocleous et al., 2022). Despite its popularity, Deformetrica necessitates the selection of at least three parameters which determine the spatial resolution of the method: two kernel sizes and one regularization parameter. Conversely, the spatial resolution of our non-rigid registration algorithm is influenced by only one regularization parameter (*γ*). Another salient feature of our method is its multi-scale nature. Our experiments have demonstrated that, for the successful alignment of two distinct shapes, it is crucial to initiate the registration process with large-scale geometrical features and progressively refine smaller details (a comparison of the results of the two methods is shown in Figure 8). Our algorithm also exhibits high robustness with respect to changes in the surface node coordinates of the target mesh, as demonstrated by the sensitivity analysis results (Supplementary Table S1). As regards the comparison between the intrinsic metrics, the improvement in generalisation of our method is mainly attributed to the enhanced parametrization of the supra-aortic vessels, which only becomes evident for higher modes. On the other hand, with regard to specificity, it is worth noting that a smaller value is more desirable. A possible explanation for our method having a higher specificity value could be the comparative method’s incorrect parametrization of the supra-aortic vessels. In fact, these small branch vessels have a high variability in terms of morphology, resulting in new shapes that may exhibit significantly different supra-aortic geometries. Therefore, if the supra-aortic vessels are correctly parametrized, random shapes would present larger variations in these regions, resulting in a decrease of the generalisation error and in an increase of the specificity value. However, it is also crucial to recognize that these three metrics (compactness, generalisation, and specificity) are intrinsic to each SSM and may not provide a comprehensive representation of the actual quality of the results Ericsson and Karlsson (2007). Thus, while our proposed method demonstrates superior performance in terms of compactness and generalisation, and slightly worse performance in specificity, an extrinsic analysis of the SSMs and their applications was made to further assess their differences. Moreover, from Supplementary Figure S1, it can be seen that there are almost negligible variations among the three metrics across different values of *α*. Although it would seem that generalisation and specificity are better for *α* = 1.0, the qualitative comparison yields intriguing insights (Supplementary Figure S2). Notably, when *α* = 1.0, the conformity of the open boundaries outweighs that of the surface (Supplementary Figure S2, shape c), resulting in an inaccurate registration of the supra-aortic vessel surface. Conversely, for certain shapes (Supplementary Figure S2, shape a), an *α* value of 0.01 proves to be inadequate to achieve an accurate registration of the supra-aortic vessels. This leads us to point out that, as already stated above (and also in the literature), these three metrics are intrinsic to the SSM and should be taken with caution when comparing SSMs created from different shapes. In conclusion, we decided to set *α* equal to 0.1 to gain a good compromise between all these considerations. When evaluating the SSMs of the entire shape (Figures 12A–C), the three metrics demonstrate similar values across the different methods. This indicates that, on the majority of the surface, the inclusion of the boundary loss and the multi-scale feature does not significantly impact the proposed method. The use of Chamfer distance as a objective function and the application of a regularized optimization process prove sufficient for addressing the correspondence problem on most of the surface, as confirmed by the qualitative comparison in Figure 11, where the surfaces exhibit a satisfactory alignment, except for branch vessels and small regions. However, when calculating the three metrics exclusively for the supra-aortic vessels (Figures 12D–F), noticeable differences arise. Notably, the proposed method without the boundary loss term (green line) demonstrates distinctly higher values for the compactness and generalisation metrics. This is also evident in the qualitative comparison of Figure 11, where the absence of the boundary loss term leads to incorrect matching of several branch vessels. Lastly, we emphasize once again the need for caution when interpreting these three metrics, as they are inherent to the Statistical Shape Model (SSM) and may not fully represent the true quality of the SSMs when they are built on different shapes. In this work, we have also developed an automatic workflow to obtain a SSM without bias often associated with the segmentation process. Our procedure allows the removal of artifacts from the shapes that a SSM should not model. The selection of predetermined lengths is attributable to technical motivations with the aim of standardizing the dataset concerning vessel length to accommodate the shortest vessels. Nonetheless, for datasets exhibiting greater heterogeneity in terms of age and size, such as those including pediatric patients or adults with a wide range of heights (our dataset only has 1.73 ± 0.08 m), this selection could be refined by considering lengths normalized with respect to each shape’s dimensions. Moreover, the proposed homogenization process is entirely automatic and this is important since the results will not depend on any manual operations performed by the user. The selection of the initial template geometry (needed for the homogenization and the non-rigid registration phases) is the only user-dependent choice. However, due to the iterative process, this dependence is highly suppressed. To assess the robustness of the methodology, we repeated the whole process two times by considering a different template geometry qualitatively similar to the average shape. Very small differences were encountered in the final average template (less than 0.1 mm). In this study, a predetermined number of fixed iterations was set as hyper-parameter to ensure convergence of the loss function for every geometry within the *homogeneous dataset*. Further improvements could include the optimization of this number of iterations at each single scale. This will result in a variable number of iterations for each scale that, for example, terminates when the maximum vertex displacement in the source mesh falls below a specified threshold. In our work, neither geometrical nor topological simplifications are assumed. The vessels are treated as fully-3D surface meshes, with respect to other works in which only the centerline coordinates and maximum radii are considered. Despite this, our SSM can explain the overall geometrical variation within the dataset with fewer modes than other works. In Bruse et al. (2017), 90% of the variance was explained with 19 modes, while in Sophocleous et al. (2019) 72% of the variance was explained with nine modes. In our work, the corresponding amounts of variance were explained with seven and two modes, respectively (see Figure 9). This can be related to several reasons. The dimensionality reduction phase is greatly affected by the nature of the analyzed dataset in terms of geometrical variations. In our dataset, in addition to healthy subjects, only aneurysmatic aortas previous to surgical intervention have been considered. In Bruse et al. (2017), they considered post-surgical aortas which were affected by two different pathologies, as well as healthy control subjects. The size of the dataset is also an important factor. A wider cohort of patients brings a potentially larger anatomical variability, which will need a greater number of modes. In Bruse et al., the dataset was composed of a total of 60 subjects, while in Sophocleous et al. (2019) 108 patients were considered. In our dataset a cohort of 47 patients was considered. Another possible reason that could explain the smaller number of modes could be the different homogenization procedure (automatic clipping) of our work. Clipping each vessel at specified lengths given by the centerline could substantially reduce the spurious geometrical variations of the shapes. Future work could enhance this study by enlarging the analyzed cohort of patients in terms of number of healthy and aneurysmatic subjects as well as by including new pathologies such as coarctations and pseudo-aneurysms. This would improve the generalisation capabilities of the SSM, while reducing errors in reconstructing new shapes (Figure 9). Moreover, the power of the SSM as a generative model of new shapes could be exploited to investigate the correlation between the morphological features and the related hemodynamic indices calculated through computational fluid dynamics simulations. In our case, for example, the study of shapes resulting from the variation of the first mode would lead to a comparative analysis of fluid dynamics in healthy and aneurysmatic aortas. Alternatively, variations in other modes would rather highlight other anatomical variations related in turn to different hemodynamic features. Finally, different non-linear dimensionality reduction methods could be explored as substitutes for PCA. In this context, Kernel PCA (Wang, 2012) or Deep auto-encoders (Hinton and Salakhutdinov, 2006) are two possible alternatives, since they could represent shape variations in more compact ways by exploiting non-linear transformations.

## Data Availability

The original contributions presented in the study are included in the article/Supplementary Material, further inquiries can be directed to the corresponding author.

## Supplementary material

Supplementary Video S1 Non-rigid registration process between two different aortic shapes: the template (source) and another random sample (target) of the homogeneous dataset. The left and top-right panels contain the source and the target displayed as the white and red surfaces, respectively. The changes of resolution of the source shape can be noticed in the left panel where the edges of the mesh are marked in black. The lower-right panel presents the evolution of the loss function throughout the registration process.

Supplementary Video S1 Non-rigid registration process between the template aorta (source) and a random sample (target) of the homogeneous dataset, compared across three different versions of the proposed approach. In each registration, the source and target shapes are displayed as white and red surfaces, respectively. The left panel represents the proposed full non-rigid registration algorithm. The central panel represents the proposed method without the boundary loss term. Finally, the right panel shows the proposed algorithm without the multi-scale feature (see section 3.3 of the paper for more details).
