# Supplementary material for: Enabling supra-aortic vessels inclusion in statistical shape models of the aorta: a novel non-rigid registration method
Source: Front Physiol. 2023 Aug 10;14:1211461. doi: 10.3389/fphys.2023.1211461 (PMC10450506; doi:10.3389/fphys.2023.1211461)
Supplement: Supplementary file 1 [file DataSheet1.pdf]

Supplementary Material

1 SENSITIVITY STUDY ON BOUNDARY LOSS COEFFICIENT

In this section, the effect of different α_j values on the generalisation, specificity and compactness metrics is reported. The sensitivity analysis was made by setting α_j equal to 0.01, 0.1 and 1.0. Figure S1 shows compactness, generalisation and specificity values for the three different α values on both the whole aorta (Figure S1(a-c)) and the supra-aortic vessels only (Figure S1(d-f)).

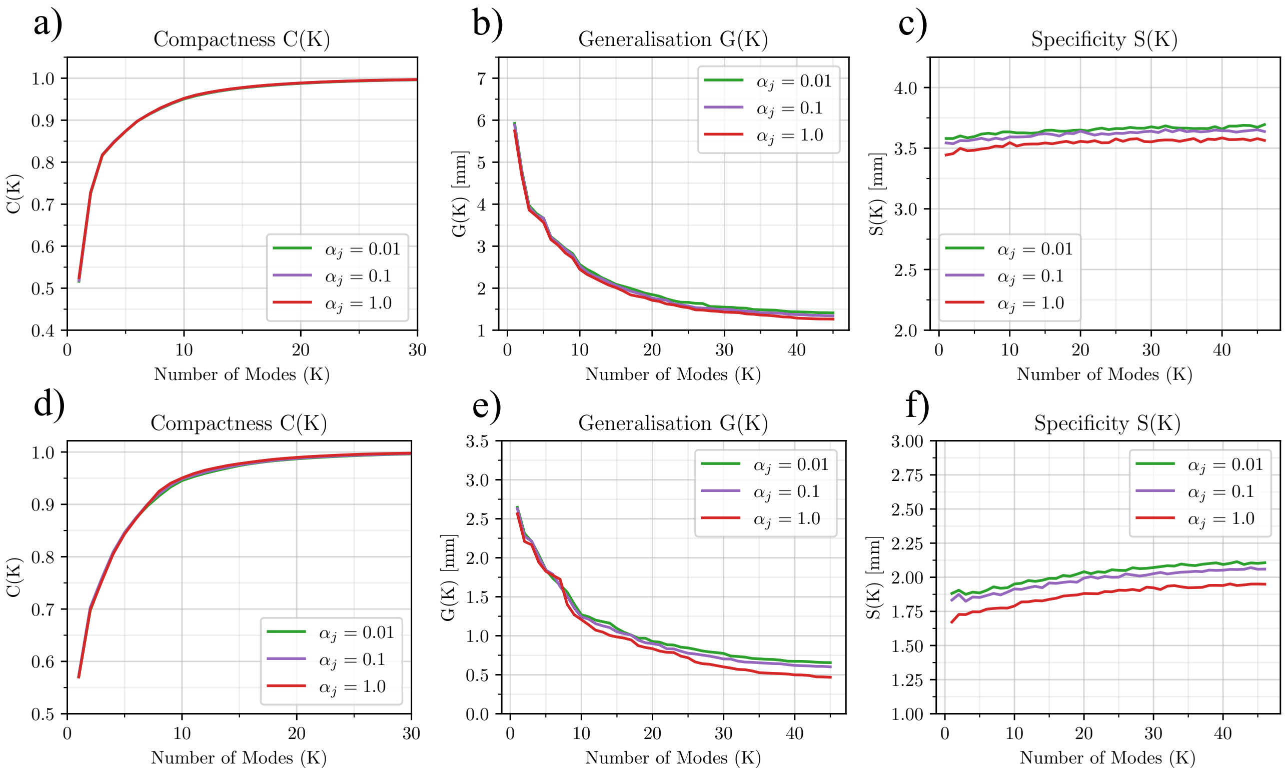

Figure S1. Comparison of the SSM metrics between our method with three different boundary loss coefficient $\alpha_j = 0.01$, $\alpha_j = 0.1$ and $\alpha_j = 1.0$ for the whole surface aorta (a-c), and for only the supra-aortic vessels (d-f).

Figure S2 shows the results of the qualitative analysis considering $\alpha_j = 0$, $\alpha_j = 0.01$, $\alpha_j = 0.1$ and $\alpha_j = 1.0$. It is worth to notice that the results for $\alpha = 0$ account for the algorithm without the open boundaries constraint.

2 SENSITIVITY STUDY ON TARGET GEOMETRY

The robustness of the algorithm was evaluated with a stability analysis by introducing small random perturbations to the surface node coordinates of the same target geometry. To perform this analysis, given a source and a target mesh, $\mathcal{M}_s$ and $\mathcal{M}_t$ respectively, $M = 10$ new discretizations of the target mesh, denoted as $\mathcal{M}_t^\tau$ for $\tau = 1, ..., M$, were created by applying a remeshing process. Subsequently,

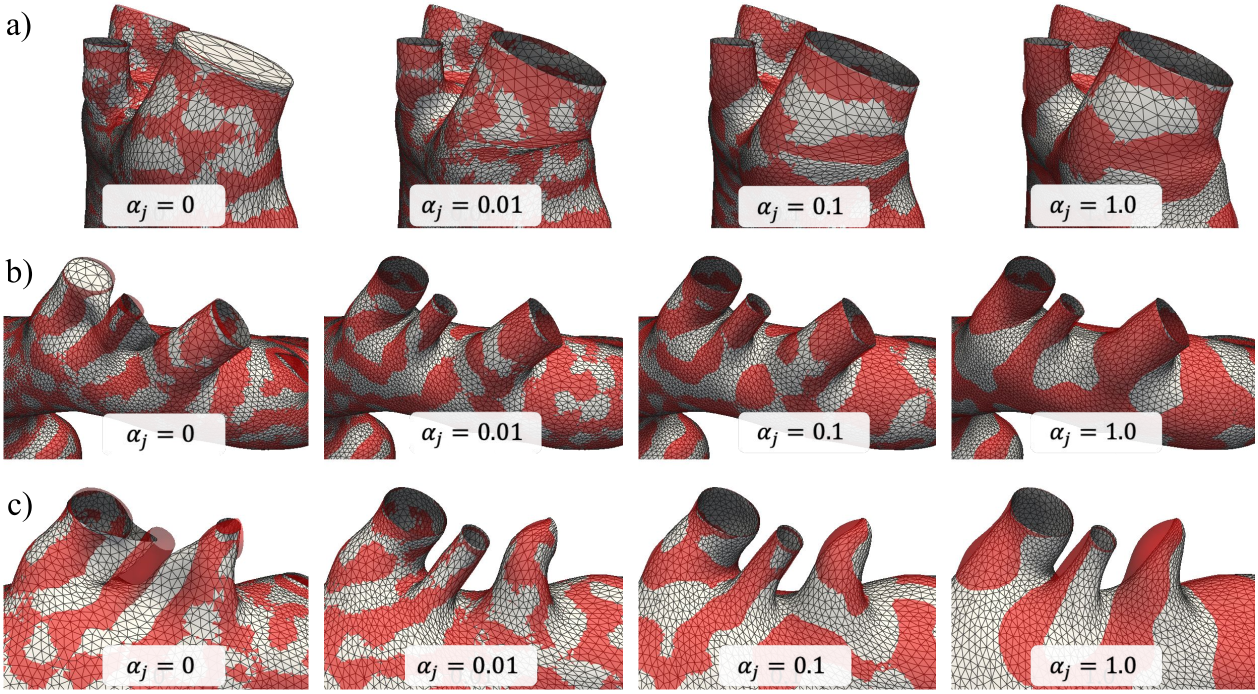

Figure S2. Qualitative comparison of different boundary loss coefficient $\alpha_j = 0, 0.01, 0.1$ and 1.0 for three different shapes.

the Chamfer and Hausdorff distances were computed between the "original" source/target registration, denoted as $\mathfrak{R}(\mathcal{M}_s, \mathcal{M}_t)$, and the "new" source/remeshed-target registration, denoted as $\mathfrak{R}(\mathcal{M}_s, \mathcal{M}_t^\tau)$, for each τ. This process was repeated four times for four different target geometries randomly chosen in the *homogeneous dataset* (shape id = $1, 2, 3, 4$). The second and third columns of Table S1 present the mean $\pm$ standard deviation of these distances for $\tau = 1, ..., 10$. Additionally, since the distances depend on the mesh resolution, the last three columns report the Chamfer and Hausdorff distances between the original target and the remeshed versions, and the average edge length $\bar{e}$ of the "original" source/target registration $\mathfrak{R}(\mathcal{M}_s, \mathcal{M}_t)$, respectively. Notably, these distances are smaller than $\bar{e}$ (last column) and very close to the initial distances between the original/remeshed target geometries (fourth and fifth columns).

shape id	$d(\mathfrak{R}(\mathcal{M}_s, \mathcal{M}_t), \mathfrak{R}(\mathcal{M}_s, \mathcal{M}_t^\tau))$		$d(\mathcal{M}_t, \mathcal{M}_t^\tau)$		$\bar{e}$ [mm]
	$(d_{CD})^{1/2}$ [mm]	d_H [mm]	$(d_{CD})^{1/2}$ [mm]	d_H [mm]	
1	0.642 ± 0.014	0.825 ± 0.028	0.647 ± 0.0012	0.721 ± 0.024	1.38
2	0.670 ± 0.001	0.854 ± 0.034	0.673 ± 0.0026	0.786 ± 0.022	1.33
3	0.670 ± 0.016	0.911 ± 0.037	0.748 ± 0.0031	0.824 ± 0.027	1.19
4	0.689 ± 0.016	0.857 ± 0.038	0.748 ± 0.0023	0.878 ± 0.039	1.19

Table S1. Chamfer and Hausdorff distances computed between the original result of the registration algorithm and perturbed versions by remeshing the target geometry. The stability analysis has been computed for 4 random shapes by remeshing each one $M = 10$ times.
